# Discovery of psoralen as a quorum sensing inhibitor suppresses *Pseudomonas aeruginosa* virulence

**DOI:** 10.1007/s00253-024-13067-9

**Published:** 2024-02-19

**Authors:** Fulong Wen, Yi Wu, Yang Yuan, Xiting Yang, Qiman Ran, Xiongyao Gan, Yidong Guo, Xinrong Wang, Yiwen Chu, Kelei Zhao

**Affiliations:** Antibiotics Research and Re-evaluation Key Laboratory of Sichuan Province, School of Pharmacy, Chengdu University, No. 2025, Chengluo Avenue, Chengdu, 610106 Sichuan China

**Keywords:** Traditional Chinese Medicine, *Pseudomonas aeruginosa*, Psoralen, Quorum sensing inhibitor, Functional profiling, Drug combination

## Abstract

**Abstract:**

*Pseudomonas aeruginosa* is a common opportunistic pathogen with growing resistance and presents heightened treatment challenges. Quorum sensing (QS) is a cell-to-cell communication system that contributes to the production of a variety of virulence factors and is also related to biofilm formation of *P. aeruginosa*. Compared to traditional antibiotics which kill bacteria directly, the anti-virulence strategy by targeting QS is a promising strategy for combating pseudomonal infections. In this study, the QS inhibition potential of the compounds derived from the Traditional Chinese Medicines was evaluated by using in silico, in vitro, and in vivo analyses. The results showed that psoralen, a natural furocoumarin compound derived from *Psoralea corylifolia* L., was capable of simultaneously inhibiting the three main QS regulators, LasR, RhlR, and PqsR of *P*. *aeruginosa.* Psoralen had no bactericidal activity but could widely inhibit the production of extracellular proteases, pyocyanin, and biofilm, and the cell motilities of the model and clinical *P*. *aeruginosa* strains. RNA-sequencing and quantitative PCR analyses further demonstrated that a majority of QS-activated genes in *P*. *aeruginosa* were suppressed by psoralen. The supplementation of psoralen could protect *Caenorhabditis elegans* from *P*. *aeruginosa* challenge, especially for the hypervirulent strain PA14. Moreover, psoralen showed synergistic antibacterial effects with polymyxin B, levofloxacin, and kanamycin. In conclusions, this study identifies the anti-QS and antibiofilm effects of psoralen against *P. aeruginosa* strains and sheds light on the discovery of anti-pseudomonal drugs among Traditional Chinese Medicines.

**Key points:**

*• Psoralen derived from Psoralea corylifolia L. inhibits the virulence-related phenotypes of P. aeruginosa.*

*• Psoralen simultaneously targets the three core regulators of P. aeruginosa QS system and inhibits the expression of a large part of downstream genes.*

*• Psoralen protects C. elegans from P. aeruginosa challenge and enhances the susceptibility of P. aeruginosa to antibiotics.*

**Supplementary Information:**

The online version contains supplementary material available at 10.1007/s00253-024-13067-9.

## Introduction

Since the discovery of antibiotics, countless lives have been preserved and the lifespan has been greatly prolonged through antibiotic treatment (Hutchings et al. 2019). Bacteria persist in their evolution under the pressure of survival in natural conditions. However, the evolutionary selection has been intensified owing to the improper and excessive application of antibiotics. As a result, antimicrobial resistance has become a worldwide health crisis, coinciding with a deceleration in the progress of novel antibiotic research and development (Nadeem et al. 2020). Therefore, there is an urgent need for novel antibiotics or treatment strategies to effectively counteract the challenge of drug resistance.

*Pseudomonas* *aeruginosa* is a Gram-negative opportunistic pathogen, widely existing in both environment and host. It frequently instigates infections in immunocompromised patients, leading to ailments such as respiratory system, blood circulation system, and urinary system infections (Qin et al. 2022; Reynolds and Kollef 2021). Currently, carbapenem-resistant *P*. *aeruginosa* has been classified as a seriously dangerous pathogen by the World Health Organization (WHO) (Willyard 2017). The resistance mechanisms of *P*. *aeruginosa* include intrinsic resistance (e.g., efflux pump), acquired resistance (e.g., horizontal gene transfer), and adaptive resistance (e.g., biofilm) (Cendra and Torrents, 2021; Pang et al. 2019; Qin et al. 2022; Thi et al 2020). Consequently, this underscores the imperative for novel strategies that diverge from traditional antibiotic approaches to address this challenge.

**Fig. 1:**
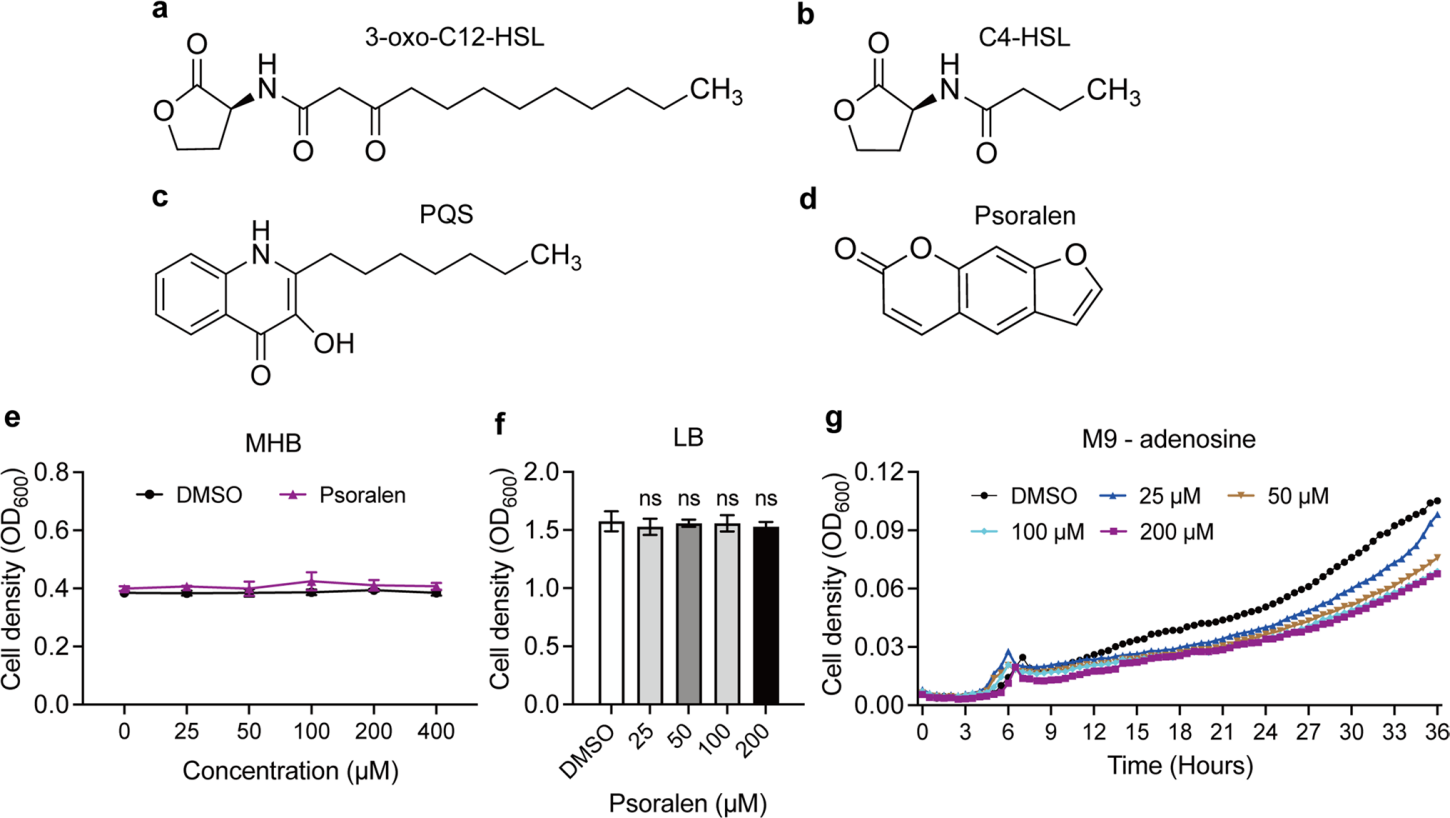
Structure of *P. aeruginosa* QS signal molecules and psoralen. Native signal molecules 3-oxo-C12-HSL (**a**), C4-HSL (**b**), PQS (**c**) of *P*. *aeruginosa* QS system. **d** Psoralen. **e** The growth of PAO1 in MHB containing different concentrations of psoralen (37 °C, 18 h). **f** The growth of PAO1 in LB broth containing different concentrations of psoralen (37 °C, 24 h). **g** The growth curves of PAO1 in M9-adenosine (0.1%, w/v) containing different concentrations of psoralen (37 °C, 36 h). The results were presented as mean ± SD and three independent experiments. The analysis method was used one-way ANOVA; ns, not significant

Quorum sensing (QS) is used to describe the process of cell-to-cell communication regulated by the density of bacterial population (Rutherford and Bassler 2012). Within *P*. *aeruginosa,* the QS system mainly consists of three components, including Las, Rhl, and PQS. Each of these components interfaces with specific natural signal molecules, also referred to as autoinducers (AIs). Specifically, N-(3-oxo-dodecanoyl)-L-homoserine lactone (3-oxo-C12-HSL) (Fig. 1a), N-butanoyl-L-homoserine lactone (C4-HSL) (Fig. 1b), and Pseudomonas Quinolone signal (PQS) (Fig. 1c) serve as the respective AIs of three components above (Lee and Zhang 2015). Upon binding of these signal molecules to their corresponding receptors, the transcription of QS genes is activated, and the expression products involve extracellular protease elastase, exotoxin, rhamnolipid, pyocyanin, and lectin A (Lee and Zhang 2015). Approximately 12% of *P*. *aeruginosa* gene expression is regulated by QS (Lin and Cheng 2019). This intricate control mechanism significantly influences a multitude of biological processes, including the formation of biofilm, the acquisition of nutrients, interspecies competition, antibiotic tolerance, and beyond (Lin and Cheng 2019; Thi et al. 2020).

The recently emerged anti-virulence strategy has garnered considerable attention due to its potential in reducing the pathogenicity of bacterial cells. This strategy revolves around targeting QS, and it exerts comparatively lesser selective pressure on survival, distinguishing it from traditional antibiotics (Defoirdt 2018). This approach primarily encompasses two facets: quorum quenching (QQ) and quorum sensing inhibitors (QSIs). QQ primarily involves the application of QS enzymes or antibodies, while QSI represents a small molecule inhibitor with enhanced stability and applicability (Fetzner 2015; Liao et al. 2022; Lu et al. 2022). Sources of QSIs can be broadly divided into natural compounds and chemical synthesized compounds, with plants serving as a crucial source of QSIs and biofilm inhibitors (Asif 2020; Chadha et al. 2022; Guzzo et al. 2020). Examples include curcumin (Rudrappa and Bais 2008), coumarin (Gutiérrez-Barranquero et al. 2015), quinic acid (Lu et al. 2021), EGCG (Hao et al. 2021), and caffeine (Chakraborty et al. 2020), which have demonstrated the ability to inhibit QS and effectively reduce the production of virulence factors and biofilm formation.

During the virtual screening process aimed at discovering anti-virulence compounds from Traditional Chinese Medicines, we directed our attention to psoralen (Fig. 1d), a natural furocoumarin compound derived from *Psoralea corylifolia* L. (Chen et al. 2023b). Psoralen exhibits a variety of effects, including the treatment of skin-related disorders, promotion of osteoblast proliferation and differentiation, antiviral, and antibacterial (Chen et al. 2023a, 2023b; Jamalis et al. 2019; Ren et al. 2020). In this study, we provided a more comprehensive understanding of the anti-QS and antibiofilm activities of psoralen and conducted an in-depth investigation into the underlying mechanism. Furthermore, the activity of psoralen in protecting *Caenorhabditis* *elegans* from *P*. *aeruginosa* infection and its synergistic antibacterial effects in combination with antibiotics were confirmed.

## Materials and methods

### Bacterial strains, media, and chemicals

The model *P*. *aeruginosa* strains PAO1 and PA14, PAO1-Δ*lasI*, PAO1-Δ*rhlI*, PAO1-Δ*pqsA*, 10 clinical strains, and the uracil nutrition-deficient *Escherichia coli* OP50 were all kept in our laboratory (Zhao et al. 2020). The used media included lysogeny broth (LB), Mueller–Hinton broth (MHB), M9 minimum growth medium with adenosine (0.1%, w/v) or casein (0.5%, w/v), M8-agar (0.5%, w/v), nematode growth medium (NGM), and peptone-glucose-sorbitol agar medium (PGS). Psoralen, with a purity of 98%, was purchased from Chengdu Alfa Biotechnology Co., Ltd. (Chengdu, China) and dissolved in dimethyl sulfoxide (DMSO) (Chron Chemicals) to a concentration of 100 mM for further usage. The QS signal molecules C12-HSL, C4-HSL, and PQS were purchased from MedChemExpress (Shanghai, China). Bacterial cells were incubated in LB broth from a single colony and cultured overnight (16–18 h) at 37 °C with shaking (180 rpm). Bacterial cells were then adjusted to optical densify of 1.0 at the absorbance of 600 nm (OD_600_ = 1.0) by using sterile saline for further usage.

### Molecular docking

Molecular docking was performed by using AutoDock 4 and Dockey, following standard protocols (Du et al. 2023). The crystal structure of LasR (PDB ID: 3IX3) and PqsR (PDB ID:6B8A) was downloaded from the RCSB PDB (https://www.rcsb.org/). RhlR (P54292) was predicted using AlphaFold (https://www.uniprot.org/). The structures of QS autoinducers 3-oxo-C12-HSL (CID: 3,246,941), C4-HSL (CID: 10,130,163) and PQS (CID: 2,763,159), and psoralen (CID: 6199) in SDF format were downloaded from PubChem (https://pubchem.ncbi.nlm.nih.gov/). These molecules were then docked to the QS proteins, and then the docking scores were compared, shedding light on their potential effectiveness as inhibitors.

### Minimum inhibitory concentration (MIC) assay

The MIC of psoralen against *P*. *aeruginosa* PAO1 was determined according to Wiegand et al. (2008) with some modifications. Briefly, *P*. *aeruginosa* PAO1 (OD_600_ = 1.0) was diluted into 100 µL of MHB in the 96-well plates, approximately 5 × 10^5^ CFU/mL. Different concentrations of psoralen were added to the wells, and the plates were incubated at 37 °C for 18 h. The same amount of DMSO was used as the control group. The absorbance was measured at 600 nm.

### Growth curve assay

Growth curve assay was determined according to Toussaint et al. (2017) with some modifications. Briefly, *P*. *aeruginosa* PAO1 (OD_600_ = 1.0) was diluted 1:100 into 200 µL of LB broth in 96-well plates with different concentrations of psoralen (0, 25, 50, 100, and 200 µM). DMSO was used as the control group. The plates were incubated at 37 °C for 24 h, measuring absorbance at 600 nm. Similarly, *P*. *aeruginosa* PAO1 was added to M9 minimum growth medium with adenosine (0.1%, w/v). The culture was incubated at 37 °C, and the absorbance at 600 nm was monitored every 30 min over a span of 36 h using SPECTROstar® Nano.

### Protease activity assay

Protease activity assay was determined according to Zhou et al. (2019) with some modifications. Briefly, *P*. *aeruginosa* PAO1 (OD_600_ = 1.0) was diluted 1:100 into 200 µL of M9 minimum growth medium with casein (0.5%, w/v) in 96-well plates with different concentrations of psoralen (0, 25, 50, 100, and 200 µM). DMSO was used as the control group. The plates were incubated at 37 °C for 24 h. Following this incubation, the absorbance at 600 nm was measured to assess protease activity.

### Pyocyanin assay

Pyocyanin assay was determined according to Essar et al. (1990). Briefly, *P*. *aeruginosa* PAO1 (OD_600_ = 1.0) was diluted 1:100 into 2 mL of LB broth in 15-mL tubes with different concentrations of psoralen (0, 25, 50, 100, and 200 µM), incubating at 37 °C with 180 rpm for 24 h. MDSO was used as the control group. After centrifugation of cultures at 12,000 rpm for 2 min, chloroform was added to the supernatant at a ratio of 5:3, fully contacted. A total of 0.2 N HCl was added to the chloroform extract at a ratio of 3:1, violently shaking. A total of 100 µL of the HCl extract was transferred to a microplate, reading at 520 nm. Furthermore, the inhibition of psoralen on the pyocyanin production of *P*. *aeruginosa* clinical isolates was measured at a concentration of 200 µM.

### Cell motility assay

Swarming, swimming, and twitching motility assays were determined according to Ha et al. (2014a), Ha et al. (2014b), and Turnbull and Whitchurch (2014), respectively. Briefly, 2 µL of *P*. *aeruginosa* PAO1 (OD_600_ = 1.0) was inoculated at the surface of the M8-agar (0.5%, w/v) plates (0.2% glucose, 0.5% casamino acids, 1 mM MgSO_4_), inoculated at the center of the LB-agar (0.3%, w/v) plates and inoculated at the bottom of the LB-agar (1%, w/v) plates, containing 200 µM of psoralen. DMSO was used as the control group. The plates were incubated at 37 °C for 18 h, after which the diameter of the motility zone was measured.

### Biofilm formation assay

Biofilm formation assay was determined according to Yuan et al. (2022). Crystal violet (CV) was used to stain biofilms attached to the walls of 96-well plates. Briefly, *P*. *aeruginosa* PAO1 (OD_600_ = 1.0) was diluted 1:100 into 200 µL of LB broth in 96-well plates with different concentrations of psoralen (0, 25, 50, 100, and 200 µM). The plates were then incubated at 37 °C for 24 h, while DMSO was used as the control group. The suspension was removed, and the wells were washed twice with PBS to remove planktonic cells. The bound biofilms were stained with 1% (w/v) CV for 10 min, after which CV was removed, and the wells were washed twice with PBS. The plates were dried, and 220 µL of 95% alcohol was used to dissolve the bound biofilms, measuring quantitatively at 595 nm. Furthermore, the inhibition of psoralen on biofilm formation of clinical isolates was measured at a concentration of 200 µM.

### Treatment of exogenous QS signal molecules

The working concentrations of QS signal molecules C12-HSL, C4-HSL, and PQS were 50 µM, and the concentration of psoralen was 200 µM. Incubating methods were the same with above. The effects of psoralen and exogenous QS signal molecules on the growth of *P*. *aeruginosa* PAO1 in M9-adenosine medium, as well as the production of virulence factors, motility, and biofilm formation were determined following the same procedures above.

### RNA sequencing

*P*. *aeruginosa* PAO1 (OD_600_ = 1.0) was diluted 1:100 into 2 mL of LB broth with or without psoralen (200 µM) and then incubated at 37 °C with shaking (180 rpm) for 20 h. Total RNAs from bacterial cells were extracted using the Animal Total RNA Isolation Kit (Foregene, Chengdu, China). RNA sequencing was conducted using the Illumina NovaSeq 6000 platform (Novogene Co., Ltd., China). For data analysis, Bowtie2 (version 2.3.4.3), HTSeq (version 0.9.1), and DESeq2 R package (version 1.20.0) were used for read mapping to the reference genome, quantification of gene expression levels, and differential expression analysis, respectively. Gene Ontology (GO) and Kyoto Encyclopedia of Genes and Genomes (KEGG) enrichment analysis were implemented using the clusterProfiler R package (version 3.8.1). A corrected *P* value of less than 0.05 was considered significant. The upregulated or downregulated genes were compared to the previously reported QS-activated genes list using Venn Diagrams (https://bioinfogp.cnb.csic.es/tools/venny/index.html) (Schuster et al. 2003).

### Quantitative PCR

The total RNAs from bacterial cells were extracted using the Animal Total RNA Isolation Kit (Foregene, Chengdu, China). qPCR was performed using the CFX Connect Real-Time PCR Detection System to detect the expression of QS genes, including *lasR*, *lasB*, *rhlR*, *rhlA*, *pqsR*, *pqsA*, *pslA*, *pilV*, *hcnA*, and *phzA1* (Table S1). The *16S* rRNA was used as an internal reference gene. The relative expression levels of genes were calculated using the 2^−ΔΔCt^ method (Zhao et al. 2018). Moreover, the QS genes of clinical isolates treated with psoralen were detected by qPCR.

### Target verification assay

The C12-HSL negative isolate PAO1-Δ*lasI* was employed to test the binding of psoralen to LasR, the C4-HSL negative isolate PAO1-Δ*rhlI* was used to test the binding of psoralen to RhlR, and the PQS negative isolate PAO1-Δ*pqsA* was utilized to test the binding of psoralen to PqsR (Rutherford and Bassler 2012). In the assay, four groups were established: (a) no treatment for mutant strains, (b) treatment of exogenous QS signal molecules (50 µM), (c) treatment of psoralen (200 µM), and (d) treatment of both exogenous QS signal molecules and psoralen. The expression levels of downstream genes *lasB*, *rhlA*, and *phzA1* which were regulated by the three QS regulators were detected by qPCR.

### Antibiotic-psoralen combination assay

The MICs of antibiotics on *P*. *aeruginosa* PAO1 and clinical isolates were determined by the broth dilution method (Wiegand et al. 2008). The antibiotic-psoralen combination assay was performed as previously reported with some modifications (Rezzoagli et al. 2020; Zhang et al. 2021). Briefly, bacterial cells (OD_600_ = 1.0) were diluted into 100 µL of MHB in 96-well plates, approximately 5 × 10^5^ CFU/mL. Different concentrations of antibiotics with or without psoralen (200 µM) were added. The plates were incubated at 37 °C for 18 h and recorded at 600 nm. The growth curves of psoralen with antibiotics were tested using continuous detection. Briefly, specific sub-MICs of antibiotics (polymyxin B, 1 µg/mL; levofloxacin, 1 µg/mL; kanamycin, 6 µg/mL) were combined with different concentrations of psoralen (50, 100, and 200 µM) in 200 µL of MHB, and the mixture was incubated at 37 °C. The absorbance at 600 nm was recorded every 1 h for 18 h using SPECTROstar ® Nano.

### *Caenorhabditis elegans* killing assay

*C. elegans* killing assay was determined according to Kirienko et al. (2014). Briefly, five adult worms were placed on NGM-OP50 plates to lay eggs for 12 h. After removing the adult worms, the plates were further cultivated at 20 °C until the worms reached the L4 stage. Psoralen was added to PGS at a final concentration of 200 µM, and DMSO was used as the control group. A total of 20 µL of *P*. *aeruginosa* PAO1 or OP50 (OD_600_ = 1.0) was smeared on PGS plates containing 50 µL of 40 mg/mL 5-fluoro-2′-deoxyuridine. The plates were then incubated at 37 °C for 24 h, followed by a 20 °C incubation for 12 h. Ten worms were picked to each plate and maintained at 20 °C. Percent survival of the infected worms was examined every 12 h until all worms in the DMSO group had died. OP50 was used as the negative control. Furthermore, we also employed the hypervirulence reference strain *P*. *aeruginosa* PA14 to construct the infection model. Additionally, an antibiotic-psoralen combination was employed in *C*. *elegans* killing assay. When using clinical isolate 4–61-8, two groups were included to evaluate the protective effect: (a) kanamycin (24 µg/mL) and (b) kanamycin-psoralen combination.

### Statistical analysis

The statistical analysis and graphing were performed using GraphPad Prism v9.0.1 (San Diego, CA, USA). The results were presented as mean ± SD (standard deviation). To compare different groups, one-way ANOVA, *t* test, or Log-rank (Mantel-Cox) test were used.

## Results

### Docking of psoralen to *P. aeruginosa* QS receptor proteins

In this study, we conducted the virtual screening of 68 compounds from 44 Sichuan Traditional Chinese Medicines, and psoralen, a furocoumarin compound derived from *P*. *corylifolia* L., was found to be capable of simultaneously docking to the three regulatory proteins, LasR, RhlR, and PqsR of* P*. *aeruginosa* QS system. The results of molecular docking predicted that the binding affinity of LasR and RhlR with psoralen was − 8.02 kcal/mol and − 6.15 kcal/mol, which were lower than those with 3-oxo-C12-HSL (− 7.80 kcal/mol) and C4-HSL (− 5.46 kcal/mol) (Table S1, Figs. S1 and S2). As shown in the docking data and the visualizations of molecules binding to LasR and RhlR, psoralen and natural ligands were similar in interacting with amino residues within the docking sites, especially van der Waals force. Though natural ligands had more H-bonds with receptors, the docking scores were higher than psoralen. It indicated that the accumulation of π-stacking between psoralen and receptors mainly attribute to stabilize the docked complexes (Table S1, Figs. S1 and S2). Moreover, the binding affinity of psoralen to PqsR was − 7.09 kcal/mol, which was higher than that of PQS (-7.62kcal/mol) (Table S1 and Fig. S3).

### Psoralen inhibits the growth of *P. aeruginosa* PAO1 in QS-required medium

The anti-virulence strategy aims to reduce the pathogenicity of pathogens without exerting survival pressure. As shown in Fig. 1e, the MIC of psoralen on *P*. *aeruginosa* PAO1 was more than 400 µM. The growth of *P*. *aeruginosa* PAO1 in LB broth was not inhibited by the maximum working concentration of psoralen (200 µM) in this study (Fig. 1f). Previous study had confirmed that the rapid growth of *P*. *aeruginosa* in the medium with adenosine as the sole carbon source was dependent on the activation of QS-controlled *nuh* gene, producing purine nucleosidase Nuh to metabolize adenosine (Toussaint et al. 2017). Here, we identified that the growth of *P*. *aeruginosa* PAO1 in M9-adenosine medium was inhibited by psoralen in a dose-dependent manner (Fig. 1g). This result indicated that psoralen exhibited an inhibitory effect on *P*. *aeruginosa* PAO1 QS system.

### Psoralen inhibits the production of virulence factors on *P. aeruginosa*

**Fig. 2:**
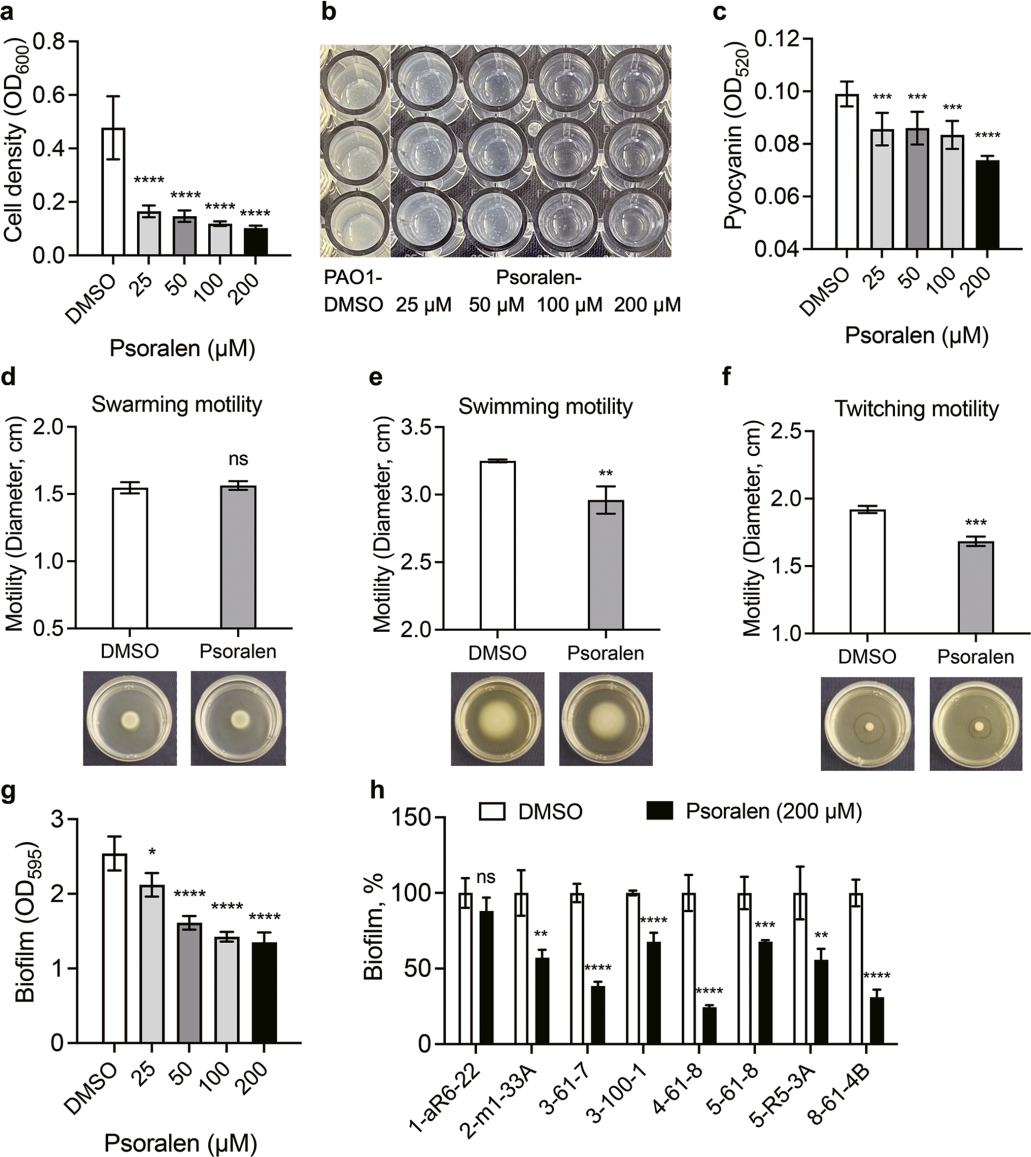
Effects of psoralen on phenotypes of *P. aeruginosa*. **a** The growth of PAO1 in M9-casein (0.5%, w/v) medium containing different concentrations of psoralen. **b** The image of growth of PAO1 in M9-casein (0.5%, w/v) medium. **c** The pyocyanin production of PAO1 in LB broth containing different concentrations of psoralen. The effects of psoralen (200 μM) on motility of PAO1 (**d**–**f**). **d** Swarming motility. **e** Swimming motility. **f** Twitching motility. The effects of psoralen on biofilm formation of *P. aeruginosa* (**g**,** h**). **g** The effect on PAO1 biofilm formation. **h** The effects on clinical isolates biofilm formation. The results were presented as mean ± SD and three independent experiments. The analysis methods were used one-way ANOVA and *t* test; ns, not significant; *P < 0.05, **P < 0.01, ***P < 0.001, ****P < 0.0001

Extracellular proteases and pyocyanin are recognized as two kinds of major virulence factors of *P*. *aeruginosa*, and their expression is regulated by QS (Das and Manefield 2012; Qin et al. 2022). In M9 minimum growth medium supplemented with casein as the sole carbon source, *P*. *aeruginosa* produces QS-controlled extracellular protease elastase to digest casein. In this study, the growth of *P*. *aeruginosa* PAO1 was significantly inhibited when cultured in M9-casein (0.5%, w/v) containing psoralen, with an inhibition rate exceeding 60% (Fig. 2a, b). For pyocyanin assay, the production of pyocyanin was significantly reduced in the presence of psoralen (Fig. 2c and Fig. S4b-d, h). These inhibition effects exhibited a dose-dependence, with the concentration of 200 µM proved to be optimal. These results indicated that psoralen inhibited the production of QS-controlled virulence factors of *P*. *aeruginosa*.

### Inhibition effects of psoralen on *P. aeruginosa* motility

The motility of *P*. *aeruginosa,* including swarming, swimming, and twitching, plays a crucial role in nutrients, colonization, and biofilm formation through the use of flagellum and pili (Thi et al. 2020). In this study, we investigated the effect of psoralen on *P*. *aeruginosa* motility. As shown in Fig. 2d–f, the swimming and twitching motility were significantly suppressed by supplementation of psoralen (200 µM) compared to the DMSO control group, while swarming motility did not show any inhibition effect. These results indicated that psoralen inhibited the motility of *P*. *aeruginosa* to some extent.

### Inhibition effects of psoralen on *P. aeruginosa* biofilm formation

Biofilm as a protective shelter for *P. aeruginosa* provides defense against environmental stresses, host immunity, etc. (Thi et al. 2020). Multiple virulence factors, such as protease and pyocyanin, produced by *P*. *aeruginosa*, are induced by QS and are closely related to biofilm formation (Qin et al. 2022). Additionally, the motility of *P*. *aeruginosa* affects colonization and biofilm diffusion. We used CV staining to quantitatively evaluate the production of *P*. *aeruginosa* biofilm. As shown in Fig. 2g, the biofilm formation of *P*. *aeruginosa* PAO1 was inhibited significantly by psoralen. At a maximum working concentration of 200 µM, the inhibition rate of biofilm was close to 50%, and the inhibition effect showed a dose-dependent manner. We investigated the effect of psoralen on the biofilm of *P*. *aeruginosa* clinical isolates, and the inhibition effect was commonly evident in clinical isolates (Fig. 2h). These results indicated that psoralen effectively inhibited *P. aeruginosa* biofilm formation.

### Effects of psoralen on *P. aeruginosa* virulence-related phenotypes with the addition of exogenous QS signal molecules

**Fig. 3:**
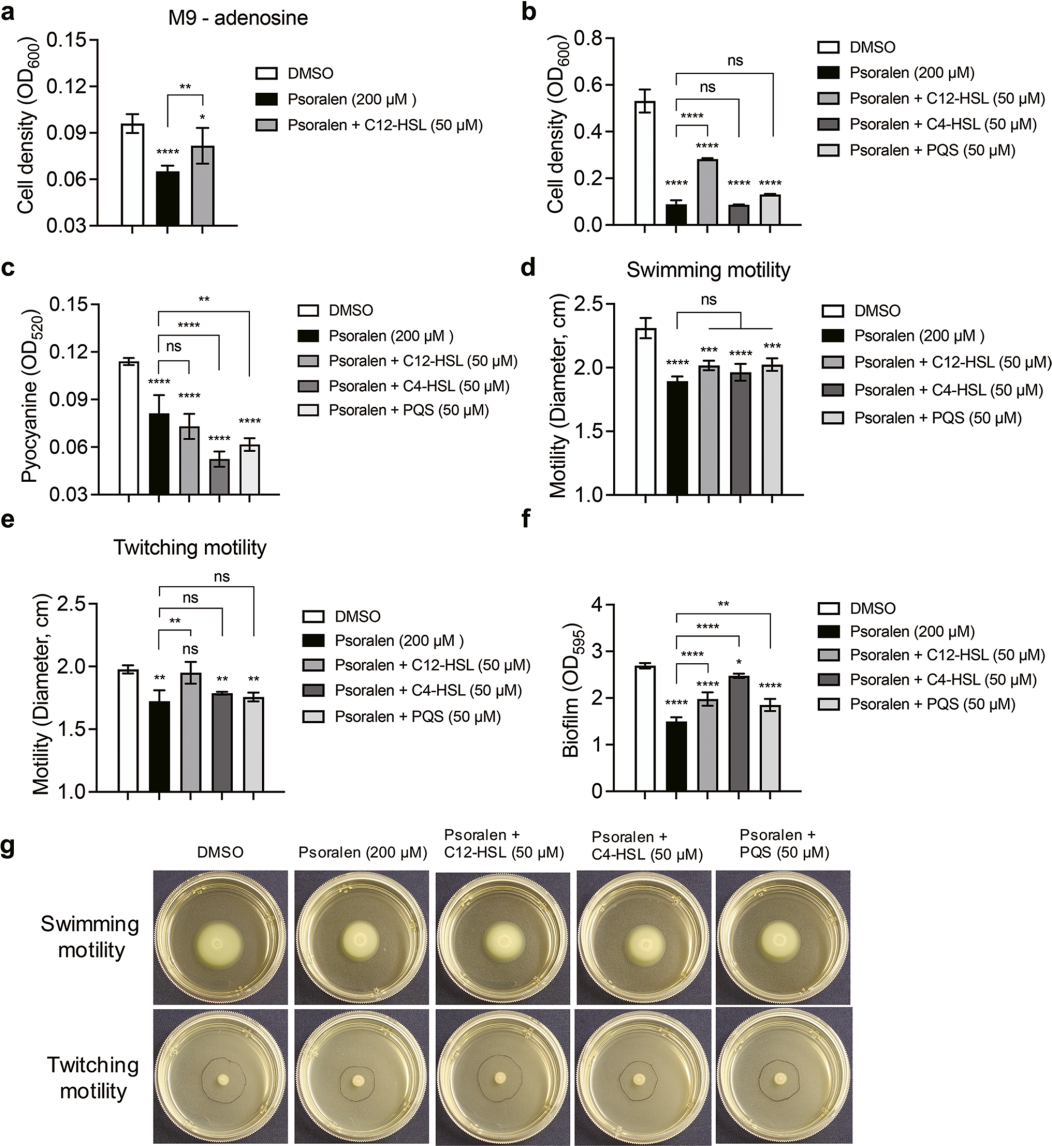
Effects of psoralen (200 μM) supplementation with AIs on phenotypic experiments of *P*. *aeruginosa*. **a** The growth of PAO1 in M9-adenosine (0.1%, w/v) medium containing psoralen supplementation C12-HSL. **b** The growth of PAO1 in M9-casein (0.5%, w/v) medium containing psoralen supplementation with different AIs. **c** The pyocyanin production of PAO1 in LB broth containing psoralen supplementation with different AIs. The effects of psoralen supplementation with different AIs on motility of PAO1 (**d**, **e**). **d** Swimming motility. **e** Twitching motility. **f** The biofilm formation of PAO1 containing psoralen supplementation with different AIs. **g** The image of motility of PAO1 containing psoralen supplementation with different AIs. The results were presented as mean ± SD and three independent experiments. The analysis method was used one-way ANOVA; ns, not significant; *P < 0.05, **P < 0.01, ***P < 0.001, ****P < 0.0001

As shown in Fig. 3a and b, the growth of *P*. *aeruginosa* PAO1 was partially rescued in the M9-adenosine and M9-casein media with the addition of exogenous C12-HSL (50 µM). The addition of the three exogenous QS signals failed to restore the production of pyocyanin of *P*. *aeruginosa* PAO1 (Fig. 3c). However, for clinical isolates, the production of pyocyanin was significantly rescued by the addition of three exogenous QS signals (Fig. S4b-d, h). For motility assay, only the group with addition of exogenous C12-HSL showed a rescue in the twitching motility of *P*. *aeruginosa* PAO1, and there were no significant changes in the others (Fig. 3d, e, g). The main reason might be that the motilities of *P*. *aeruginosa* were not directly regulated by QS. The biofilm formation was partially rescued by the addition of three exogenous QS signals, among which C4-HSL was the most significant (Fig. 3f).

### Psoralen inhibits the expression of QS-controlled genes

**Fig. 4:**
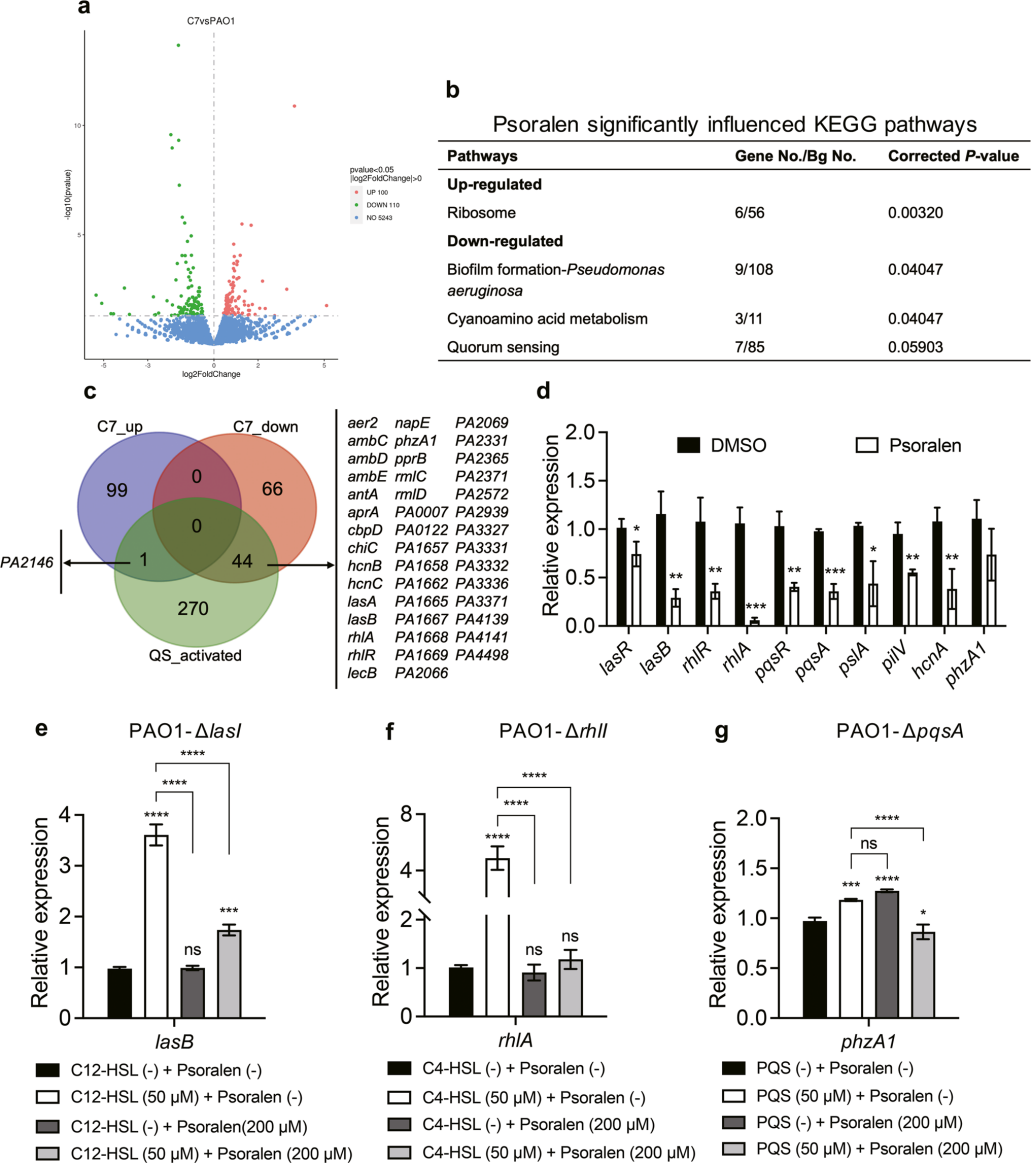
The mechanism of anti-QS and antibiofilm activity of psoralen. **a** Volcano plot of differentially expressed genes. **b** Psoralen significantly influenced KEGG pathways. **c** Venn diagram of differentially expressed genes. **d** Expression of important QS genes of psoralen-treated PAO1 was determined by qPCR. **e** The expression of *lasB* in PAO1-Δ*lasI* under different conditions. **f** The expression of *rhlA* in PAO1-Δ*rhlI* under different conditions. **g** The expression of *phzA1* in PAO1*-ΔpqsA* under different conditions. The results were presented as mean ± SD and three independent experiments. C7 indicated psoralen-treated PAO1. The analysis methods were used one-way ANOVA and *t* test, *P < 0.05, **P < 0.01, ***P < 0.001, ****P < 0.0001

RNA-sequencing was then conducted to explore the effect of psoralen on the global transcription of *P*. *aeruginosa*. The results showed that 100 genes were upregulated and 110 genes were downregulated (*P* < 0.05) in *P*. *aeruginosa* PAO1 treated with 200 µM of psoralen (Fig. 4a and Dataset S1). The KEGG pathway analysis indicated that biofilm formation-*Pseudomonas aeruginosa* and cyanoamino acid metabolism was significantly enriched among the downregulated genes (*P* < 0.05), while ribosome was significantly enriched among the upregulated genes (*P* < 0.05, Fig. 4b). Additionally, *rhlR*/*rhlA*, *lasA*, and *lasB* in QS of KEGG pathways were also enriched among the downregulated genes while the downregulation did not show significance (*P* = 0.05903, Fig. 4b). The pathway of biofilm formation-*Pseudomonas aeruginosa* is composed of four parts: CAMP/Vfr signaling pathway, QS pathway, Gac/Rsm pathway, and c-di-GMP signaling pathway. Along with typical virulence genes in quorum sensing pathway, we also observed that the expression of HSI-I-type VI secretion system was downregulated in Gac/Rsm pathway, which plays a vital role in biofilm formation and is regulated by QS. The GO terms of oxidoreductase activity, cation binding, and metal ion binding were significantly downregulated (Fig. S5a), while ribosome, ribonucleoprotein complex, etc. were significantly upregulated (Fig. S5b). These results suggested that psoralen affected the metabolic process of *P*. *aeruginosa*, especially biofilm formation and other secondary metabolic activities.

To further investigate the relevancy of the significantly changed genes with QS, we compared them to the list of QS-activated genes (Schuster et al. 2003). Among the 110 downregulated genes, 44 were found to be associated with QS (Fig. 4c). Subsequently, we detected the expression levels of several crucial QS genes using qPCR, including *lasR*, *lasB*, *rhlR*, *rhlA*, *pqsR*, *pqsA*, *pslA*, *pilV*, *hcnA*, and *phzA1*. Figure 4d shows the expression levels of QS regulatory genes and downstream functional genes were inhibited, and the Rhl system was the most obviously suppressed which was consistent with RNA sequencing. Similar results were detected in clinical isolates (Fig. S4e-g). Thus, these results provided further validation of the phenotypic inhibition effects. Combining phenotypic results with RNA sequencing, these results reaffirmed that psoralen inhibited QS and reduced the production of virulence factors of *P*. *aeruginosa.*

### Psoralen targets the three QS regulators of *P. aeruginosa*

The signal-negative mutant strains Δ*lasI* (C12-HSL-negative), Δ*rhlI* (C4-HSL-negative), and Δ*pqsA* (PQS-negative) were used to assess the interaction of psoralen with the three QS regulators. The expression levels of *lasB*, *rhlA*, and *phzA1* were detected by qPCR, as their expressions were regulated by LasR, RhlR, and PqsR, respectively. As shown in Fig. 4e, the expression of *lasB* increased to about threefold with the addition of exogenous C12-HSL, while its expression was significantly downregulated with the addition of both exogenous C12-HSL and psoralen. Similar results were detected in Δ*rhlI* mutant strain, where the expression of *rhlA* increased to about fourfold with the addition of exogenous C4-HSL, and the inhibition of *rhlA* expression was more pronounced with the addition of both exogenous C4-HSL and psoralen (Fig. 4f). These results suggested that psoralen could competitively inhibit the interactions of C12-HSL/C4-HSL to LasR/RhlR, thereby inhibiting the expressions of downstream genes. These findings were consistent with the virtual screening, as psoralen-LasR/RhlR had a higher affinity than C12-HSL/C4-HSL-LasR/RhlR (Table S1). Moreover, the expression of *phzA1* in Δ*pqsA* mutant strain was increased by the treatment of PQS, further supplementation of psoralen could also significantly inhibit the expression of *phzA1*, albeit the expression of which was promoted by the treatment of psoralen alone (Fig. 4g). This result indicated that PqsR was also the target of psoralen, although the regulation of *pqs-*QS system was much more complicated than those of *las-* and *rhl-*QS systems (Lee and Zhang 2015), and the results of virtual screening also predicted that psoralen-PqsR exhibited a lower affinity than PQS-PqsR (Table S1).

### Synergistic antibacterial effects of psoralen with antibiotics

**Fig. 5:**
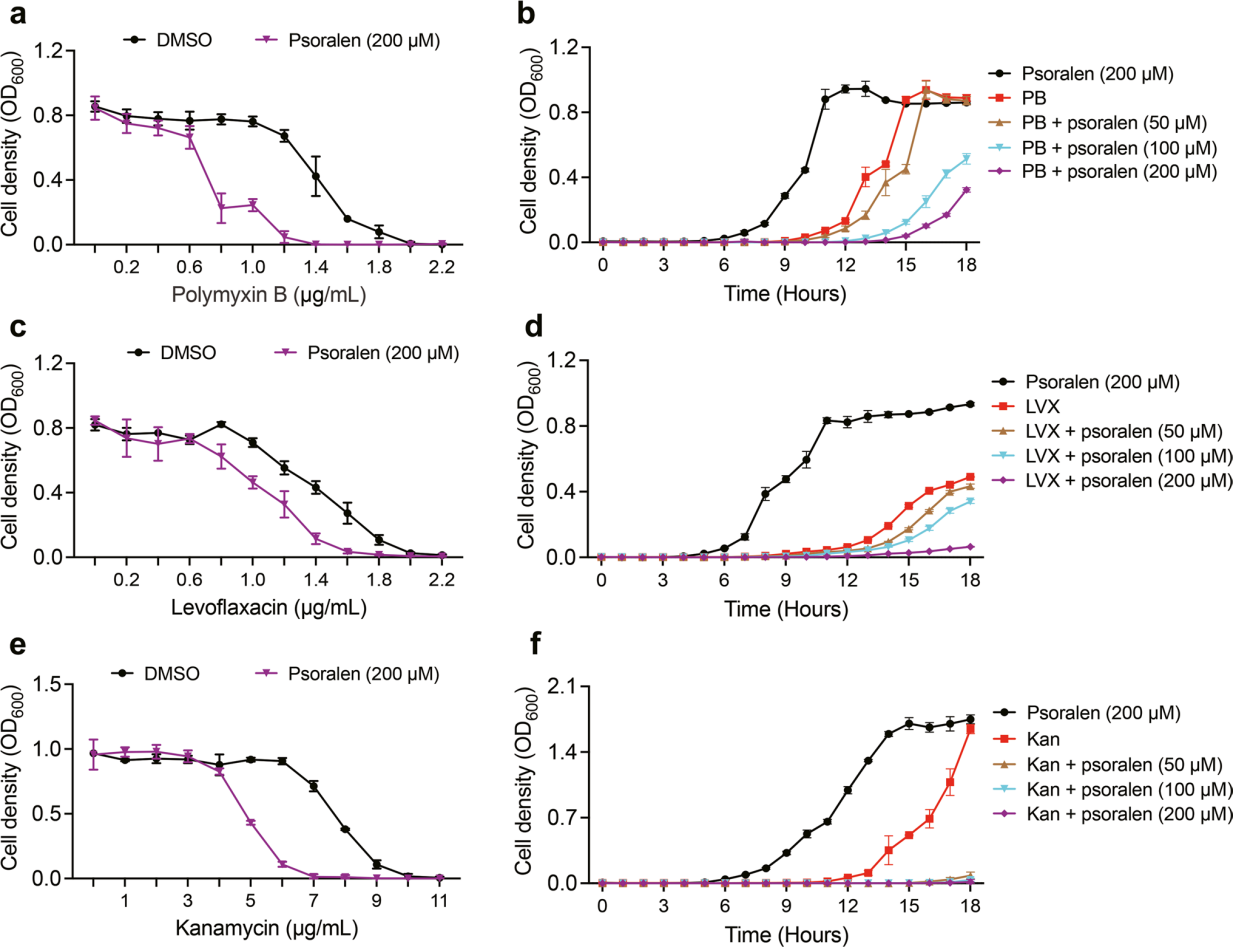
Effects of antibiotic-psoralen combinations on growth of PAO1 and clinical isolates. Effects on growth of PAO1 and clinical isolates combining antibiotics with psoralen (**a**, **c**, **e**). The growth curves of PAO1 and clinical isolates combining sub-MICs with different concentrations of psoralen (**b**, **d**, **f**). **a** PAO1. **b** PAO1, and polymyxin B (PB): 1 μg/mL. **c** Clinical isolate 3–100-1. **d** Clinical isolate 3–100-1, and levofloxacin (LVX): 1 μg/mL. **e** Clinical isolate 5-R5-3A. **f** Clinical isolate 5-R5-3A, and kanamycin (Kan): 6 μg/mL. The results were presented as mean ± SD and three independent experiments

We then measured the MICs of *P*. *aeruginosa* PAO1 and clinical isolates against clinically common antibiotics (Table S3), then explored the synergistic antibacterial effects of psoralen when combined with antibiotics. The results showed the susceptibility of *P*. *aeruginosa* PAO1 and clinical isolates to antibiotics was increased when psoralen was combined with polymyxin B, levofloxacin, and kanamycin (Figs. 5a, c, d and S6). For polymyxin B, at a concentration range of 0.6–2.0 µg/mL, the supplementation of psoralen (200 µM) significantly increased the antibacterial effect on *P*. *aeruginosa* PAO1 (Fig. 5a). A dose-dependent inhibition effect on the growth of *P*. *aeruginosa* PAO1 when sub-MIC of polymyxin B (1 µg/mL) was combined with different concentrations of psoralen (50, 100, and 200 µM) (Fig. 5b). Similar synergistic antibacterial effects were observed when combining levofloxacin and kanamycin with psoralen for other isolates (Figs. 5c–f and S6). In summary, the synergistic antibacterial effect was significantly enhanced when combination psoralen with these antibiotics, which indicated a potential as an effective strategy for combating *P. aeruginosa* infections.

### Protective effect of psoralen in the *C. elegans* infection models

**Fig. 6:**
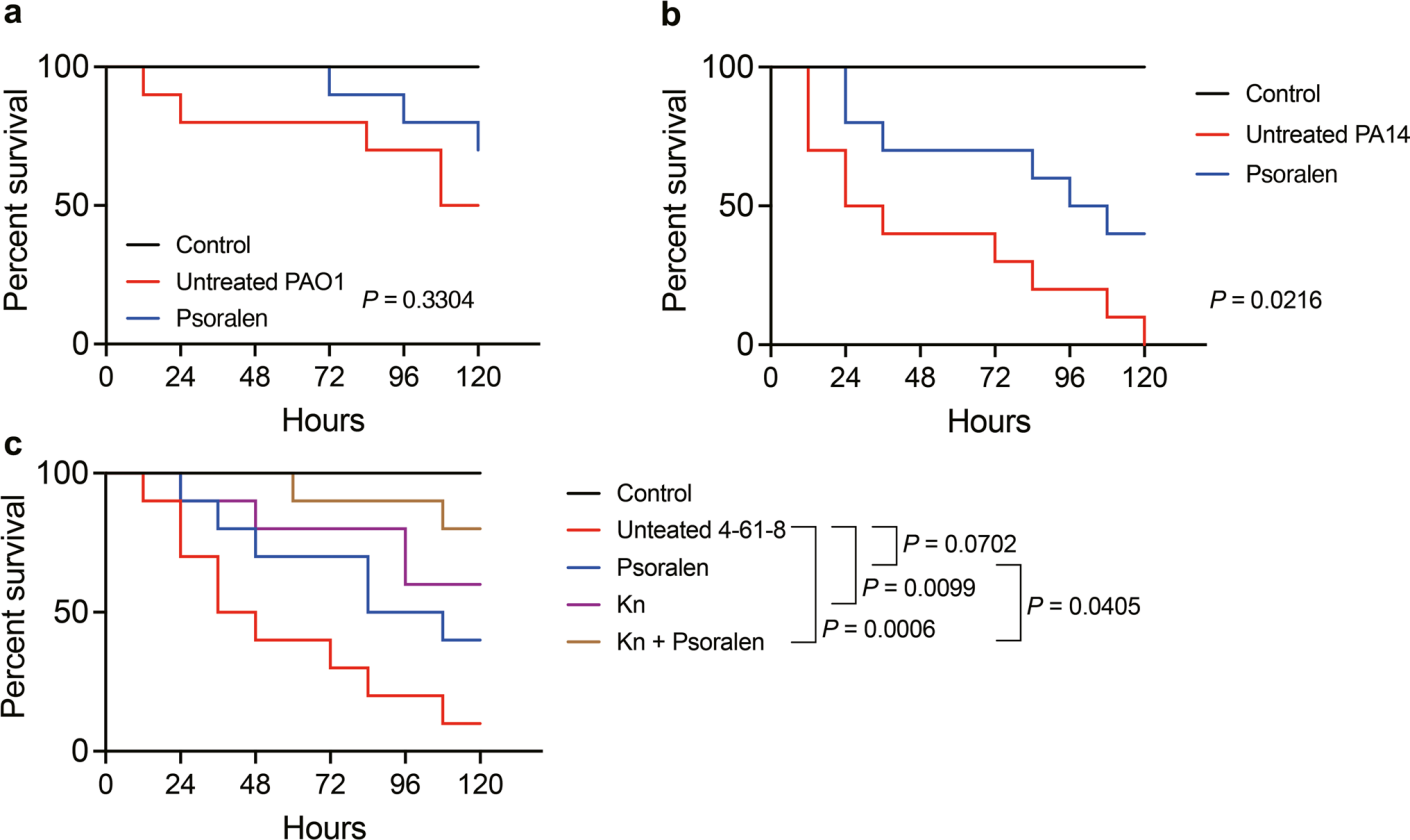
Protective effects of psoralen in *C*. *elegans* infection models. **a** PAO1-killing assay. **b** PA14-killing assay. **c** Clinical isolate *P*. *aeruginosa* 4–61-8-killing assay. The concentration of psoralen was 200 µM, and the concentration of kanamycin was 24 µg/mL. Log-rank (Mantel-Cox) test

Fast killing is a rapid toxin-mediated death induced by virulence factors produced by *P*. *aeruginosa*, including extracellular protease and pyocyanin (Kirienko et al. 2014). To investigate the protective effect of psoralen, we conducted three *C*. *elegans* infection models, including PAO1-fast killing, PA14-fast killing, and clinical isolate 4–61-8-fast killing. The results showed that psoralen displayed a protective effect to PAO1-fast killing assay but not significant (*P* = 0.3304, Fig. 6a). On the other hand, psoralen exhibited a sustained protective effect to PA14-fast killing, significantly reducing mortality compared to the untreated group (*P* = 0.0216, Fig. 6b). The antibiotic-psoralen combination was then used in the *C. elegans* infection model. As shown in Fig. 6c, in the clinical isolate *P. aeruginosa* 4–61-8-fast killing assay, the mortality rate of worms was reduced by supplementation with psoralen (*P* = 0.0702) or kanamycin (*P* = 0.0099) alone compared to the untreated group, while the mortality rate was only 20% in the combined treatment group (*P* = 0.0006). These results suggested psoralen and its combination with kanamycin protected *C. elegans* from *P. aeruginosa* infection.

## Discussion

Presently, the rise of antimicrobial resistance has emerged as a global health concern (Hutchings et al. 2019). *P. aeruginosa*, as a common opportunistic pathogen, causes various infection types (Reynolds and Kollef 2021; Qin et al. 2022). The antimicrobial resistance of *P. aeruginosa* has become more severe due to the misuse and overuse of antibiotics, as well as resistance mechanisms (Pang et al. 2019; Cendra and Torrents, 2021; Qin et al. 2022). This renders antibiotic treatment more challenging, thus underscoring the pressing need for innovative therapeutic strategies. QS is a vital signal communication system within *P*. *aeruginosa*, involving the secretion of virulence factors, such as extracellular protease elastase, rhamnolipid, and pyocyanin (Qin et al. 2022). Furthermore, the process of biofilm formation is closely connected to QS (Cendra and Torrents, 2021). Thus, targeting QS of *P. aeruginosa* is an effective way to inhibit the production of virulence factors and biofilm formation.

Kinds of compounds extracted from Traditional Chinese Medicines have been proved to exhibit anti-QS and antibiofilm effects, such as emodin (Ding et al. 2011), rhein (Ding et al. 2011), curcumin (Rudrappa and Bais 2008), and quinic acid (Lu et al. 2021). Psoralen, derived from *P. corylifolia* L., belongs to the simplest linear furocoumarins and exhibits various pharmacological activities (Chen et al. 2023b; Jamalis et al. 2019; Ren et al. 2020). Previous research has reported the inhibition of biofilm formation in *P*. *aeruginosa* by psoralen with limited elucidation of the underlying molecular mechanisms (Zeng et al. 2008). Certain furocoumarins, including bergamottin and dihydroxybergamottin, have also been reported for inhibiting biofilm formation of *P*. *aeruginosa* (Girennavar et al. 2008). Additionally, Li et al. explored the inhibition effect of psoralen on *Porphyromonas gingivalis* biofilm (Li et al. 2018). As the simplest furocoumarin compound, it is necessary to study the anti-QS and antibiofilm effects and in-depth mechanisms of psoralen, which is beneficial for the subsequent study on the anti-QS and antibiofilm effects of series furocoumarin compounds. In this study, we explored the inhibition effects of psoralen on QS and biofilm, and further investigated the possible mechanism.

In this study, psoralen demonstrated higher binding energies with LasR and RhlR than natural ligands through virtual screening, and it also exhibited the potential to bind with PqsR (Table S1 and Figs. S1-S3). These results suggested that psoralen had potential to bind three QS regulatory proteins of *P*. *aeruginosa.* The production of multiple virulence factors of *P*. *aeruginosa* was inhibited by psoralen, such as extracellular protease, pyocyanin, and motility, all of which are related to the biofilm formation (Figs. 2a–f and S4b-d, h). Moreover, psoralen effectively inhibited biofilm formation of *P*. *aeruginosa* (Fig. 2g–h). In transcriptomic analysis, KEGG pathways of biofilm formation and QS were significantly enriched and downregulated (Fig. 4b). We then performed an analysis of significantly changed genes and found that among the 110 genes which were significantly downregulated, 44 were associated with QS (Fig. 4c). As we employed further verification through qPCR and QS mutants, the related genes of the three QS systems were significantly downregulated by the supplementation of psoralen and the QS signals, and the *rhl-*QS system was the most significant (Figs. 4d–f and S4e-g). Therefore, production of virulence factors and biofilm formation could be inhibited by psoralen through targeting the QS system of *P*. *aeruginosa*.

In the treatment of exogenous QS signal molecule assay, we observed that some phenotypes associated with virulence inhibited by psoralen were rescued, including the growth of *P*. *aeruginosa* in QS-required medium, extracellular protease, and biofilm formation (Fig. 3). However, the pyocyanin production of PAO1 was more inhibited (Fig. 3c). This might be because the production of pyocyanin was mainly affected by the *rhl* and *pqs* systems, and there was a negative feedback regulation between them (Das and Manefield 2012; Qin et al. 2022). The *C*. *elegans*-*P*. *aeruginosa* killing is mainly mediated by virulence factors, such as extracellular protease and pyocyanin. Due to the inhibition effects of psoralen on the production of virulence factor of *P*. *aeruginosa*, we found that psoralen had a significant protective effect to *C*. *elegans* from *P*. *aeruginosa* (Fig. 6).

Currently, the resistance of *P*. *aeruginosa* continues to increase. According to the statistics on clinical resistance rates of *P. aeruginosa* in China from the CHINET website (http://www.chinets.com/Data/AntibioticDrugFast), for instance, the resistance rate to levofloxacin has exceeded 20%. Polymyxin B serves as the final defense in treating carbapenem-resistant bacteria, while its resistance rate increases as its use increases (El-Sayed Ahmed et al. 2020). However, antibiotic therapy remains a pivotal approach for *P*. *aeruginosa* infections. Presently, the combination of antibiotics and nonantibacterial drugs is well noted. Zhang et al. (2021) reported that the combination of colistin and furanone C-30 enhanced the antibacterial and antibiofilm activities against Gram-negative bacteria. Topa et al. (2020) also reported that the combination of colistin and cinnamaldehyde had a synergistic killing effect on *P*. *aeruginosa*. In this study, we explored the potential of combining psoralen with antibiotics. Our findings revealed that the combination of psoralen with polymyxin B, levofloxacin, and kanamycin enhanced the antibacterial effect against *P. aeruginosa* (Figs. 5 and S6). Upon interacting with antibiotics, psoralen reduced the production of multiple virulence factors and biofilm formation of *P*. *aeruginosa* through targeting QS. Notably, antibiotic resistance of *P*. *aeruginosa* is closely related to virulence factors, biofilm, secretion systems, etc., which are regulated by QS. We speculated that the observed results could stem from psoralen’s ability to inhibit QS and biofilm formation. We applied antibiotic-psoralen combination to *C*. *elegans* killing assay, and the survival rate of worms was increased compared to antibiotics or psoralen alone (Fig. 6c). Clinically, patients with chronic obstructive pulmonary disease and cystic fibrosis are affected by *P*. *aeruginosa* (Malhotra et al. 2019; Rakhimova et al. 2009), and the existence of biofilms renders complete eradication through antibiotic therapy challenging, thus suggesting a prospect for future utilization of anti-virulence drugs as a complement alongside antibiotic therapy.

Collectively, in this study, we determined that psoralen targeted QS, leading to the inhibition of virulence factor production and biofilm formation. We elaborated the underlying mechanism of psoralen’s QS inhibition and demonstrated the significant protective effect of psoralen on *C*. *elegans*. In the synergistic antibacterial assay, some references were provided for potential future investigations of combination therapies. Therefore, the study laid the groundwork for the development of furocoumarin-based anti-virulence drugs, offering valuable basics for future research in this direction.

## Supplementary Information

Below is the link to the electronic supplementary material.

Supplementary file1 (PDF 2.18 MB)

Supplementary file2 (XLS 60 KB)

## Data Availability

The datasets generated and analyzed during the current study are available in the NCBI BioProject repository and the registration number PRJNA1015373 (https://www.ncbi.nlm.nih.gov/bioproject/PRJNA1015373).
