# Supplementary material for: Discovery of psoralen as a quorum sensing inhibitor suppresses Pseudomonas aeruginosa virulence
Source: Appl Microbiol Biotechnol. 2024 Feb 19;108:222. doi: 10.1007/s00253-024-13067-9 (PMC10876730; doi:10.1007/s00253-024-13067-9)
Supplement: Supplementary file 1 [file 253_2024_13067_MOESM1_ESM.pdf]

Discovery of psoralen as a quorum-sensing inhibitor suppresses *Pseudomonas aeruginosa* virulence

Fulong Wen, Yi Wu, Yang Yuan, Xiting Yang, Qiman Ran, Xiongyao Gan, Yidong Guo, Xinrong Wang, Yiwen Chu* and Kelei Zhao*

[1] Antibiotics Research and Re-evaluation Key Laboratory of Sichuan Province, School of Pharmacy, Chengdu University, Chengdu 610106, China

* Correspondence:

Address: No. 2025, Chengluo Avenue, Chengdu 610106, Sichuan, China. Yiwen Chu, Email: chuyiwen@cdu.edu.cn, Kelei Zhao, Email: zhaokelei@cdu.edu.cn.

Supplementary Figures

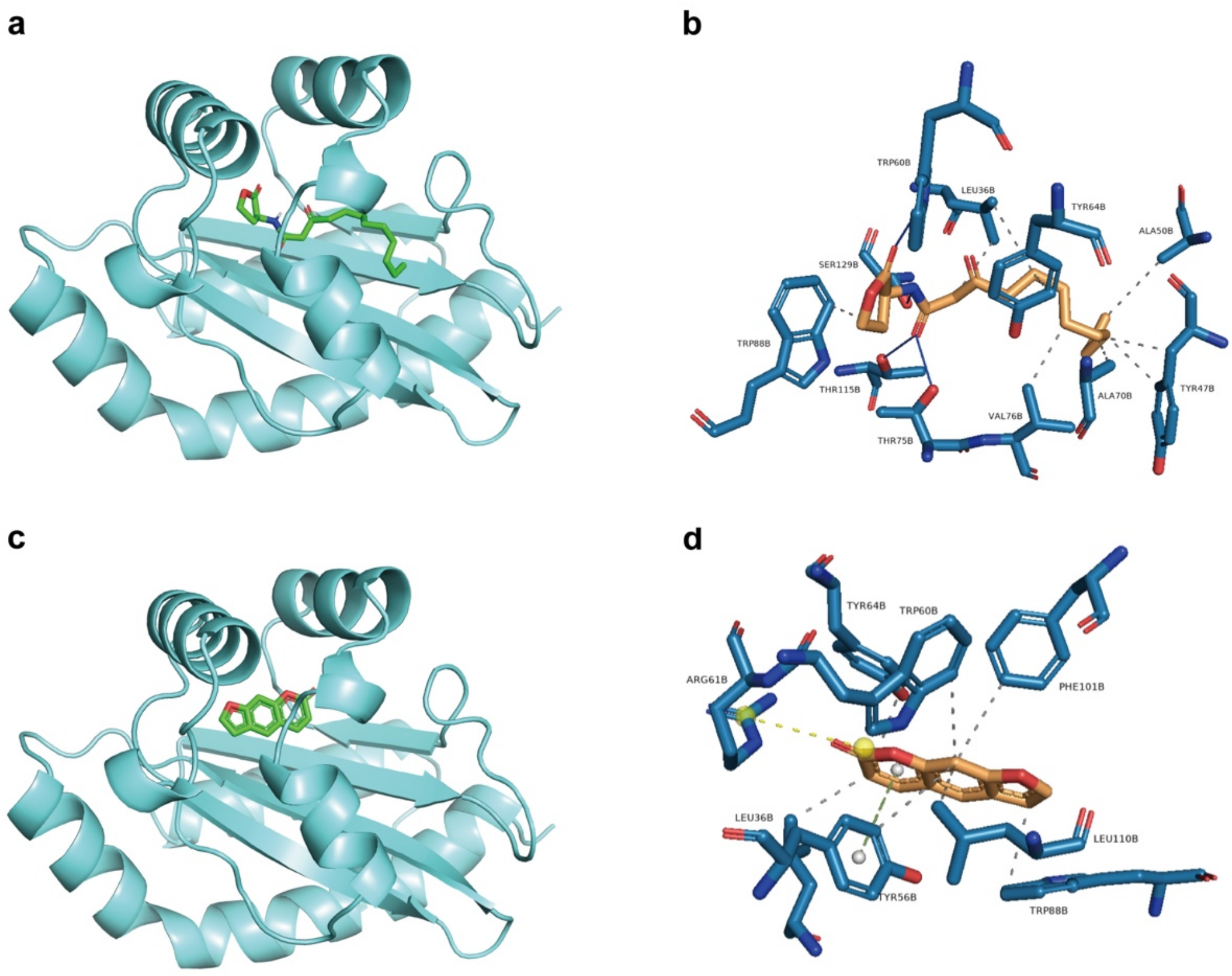

Fig. S1 Molecular docking of LasR protein binding to 3-oxo-C12-HSL (**a**, **b**) and psoralen (**c**, **d**).

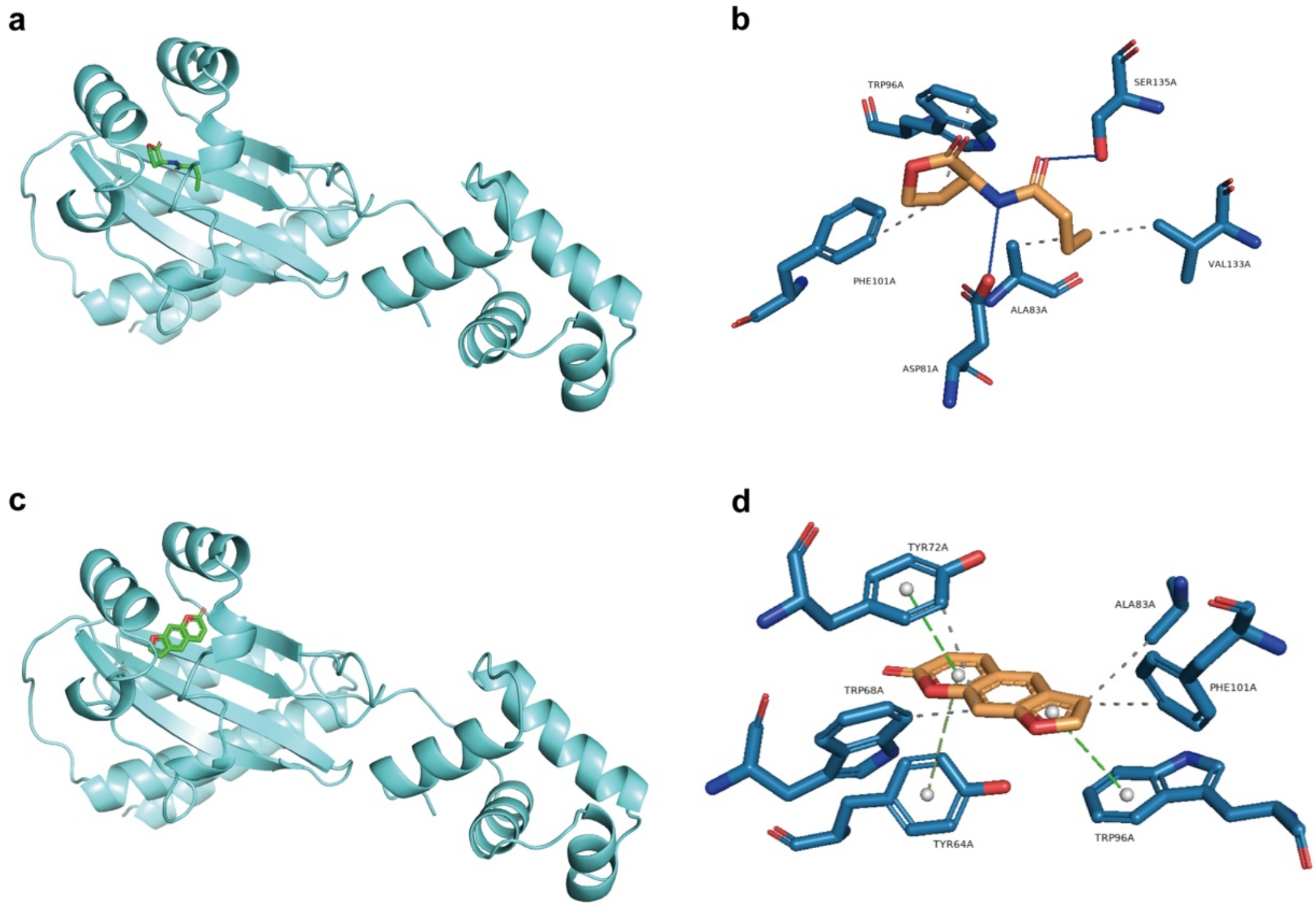

Fig. S2 Molecular docking of RhlR protein binding to C4-HSL (**a**, **b**) and psoralen (**c**, **d**).

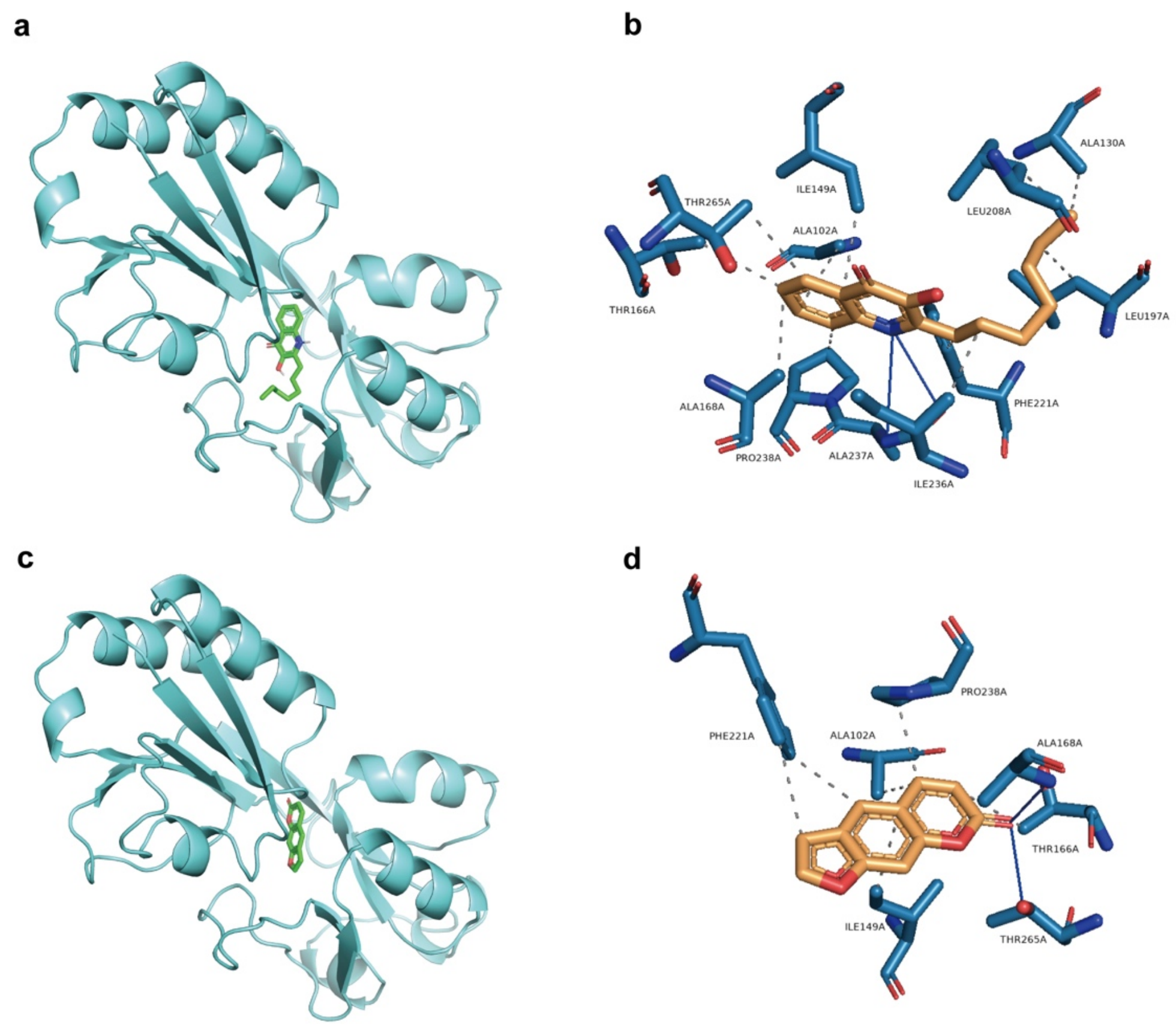

Fig. S3 Molecular docking of PqsR protein binding to PQS (**a**, **b**) and psoralen (**c**, **d**).

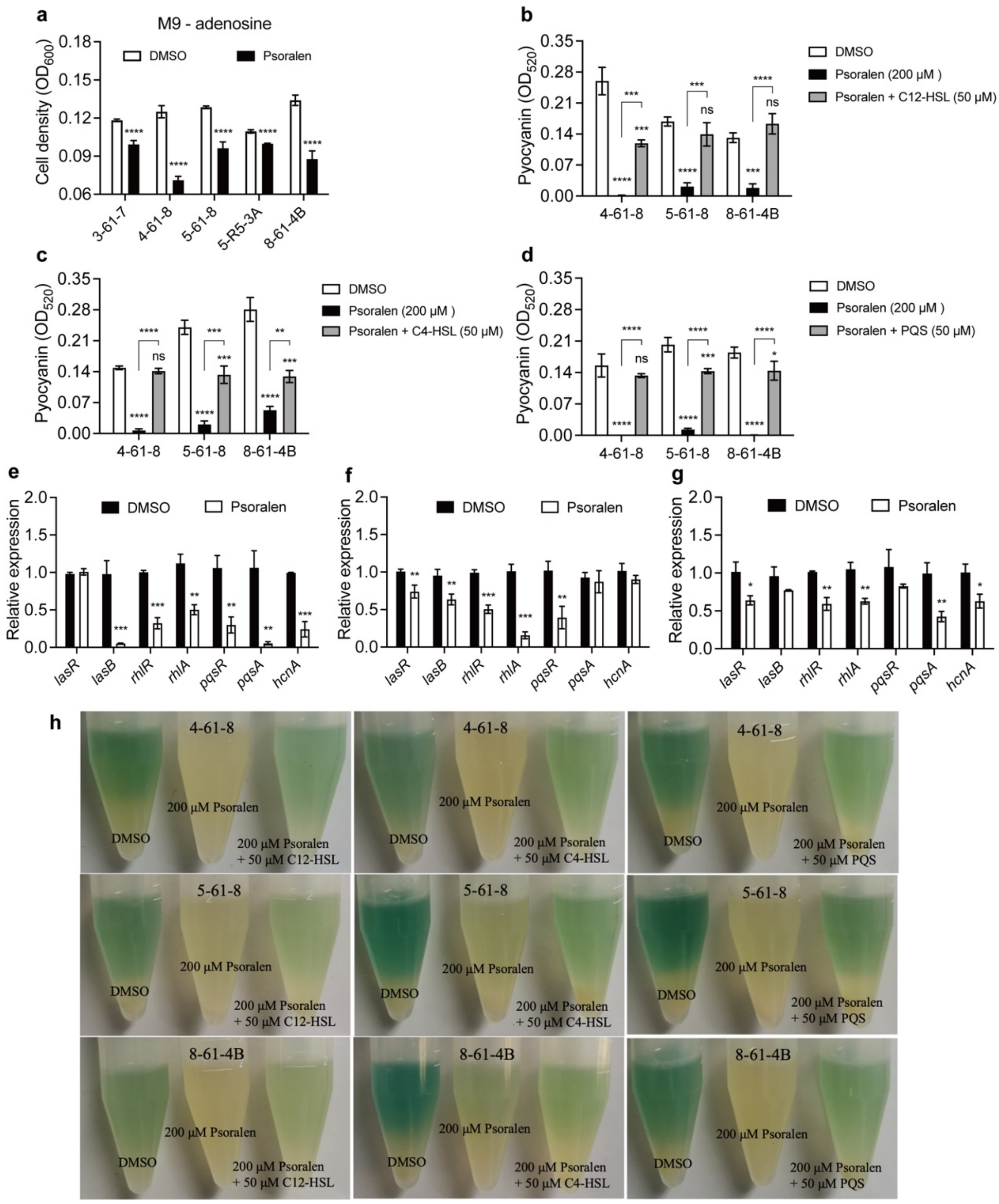

Fig. S4 Effects of psoralen on clinical isolates. **a** The growth of clinical isolates in M9-adenosine (0.1%, w/v) medium containing psoralen (200 μM). The pyocyanin production of clinical isolates in LB broth containing psoralen supplementation with different AIs (**b**-**d**). **b** Psoralen supplementation with C12-HSL. **c** Psoralen supplementation with C4-HSL. **d** Psoralen supplementation with PQS. Expression of main QS genes of psoralen-treated clinical isolates was determined by qPCR(**e**-**g**). **e** Clinical isolate 4-61-8. **f** Clinical isolate 5-61-8. **g** Clinical isolate 8-61-4B. **h** The image of pyocyanin production of clinical isolates containing psoralen supplementation with different AIs. The results were presented as mean ± SD, and three independent experiments. The analysis methods were used one-way ANOVA and t test, ns, not significant, *, $P < 0.05$, **, $P < 0.01$, ***, $P < 0.001$, ****, $P < 0.0001$.

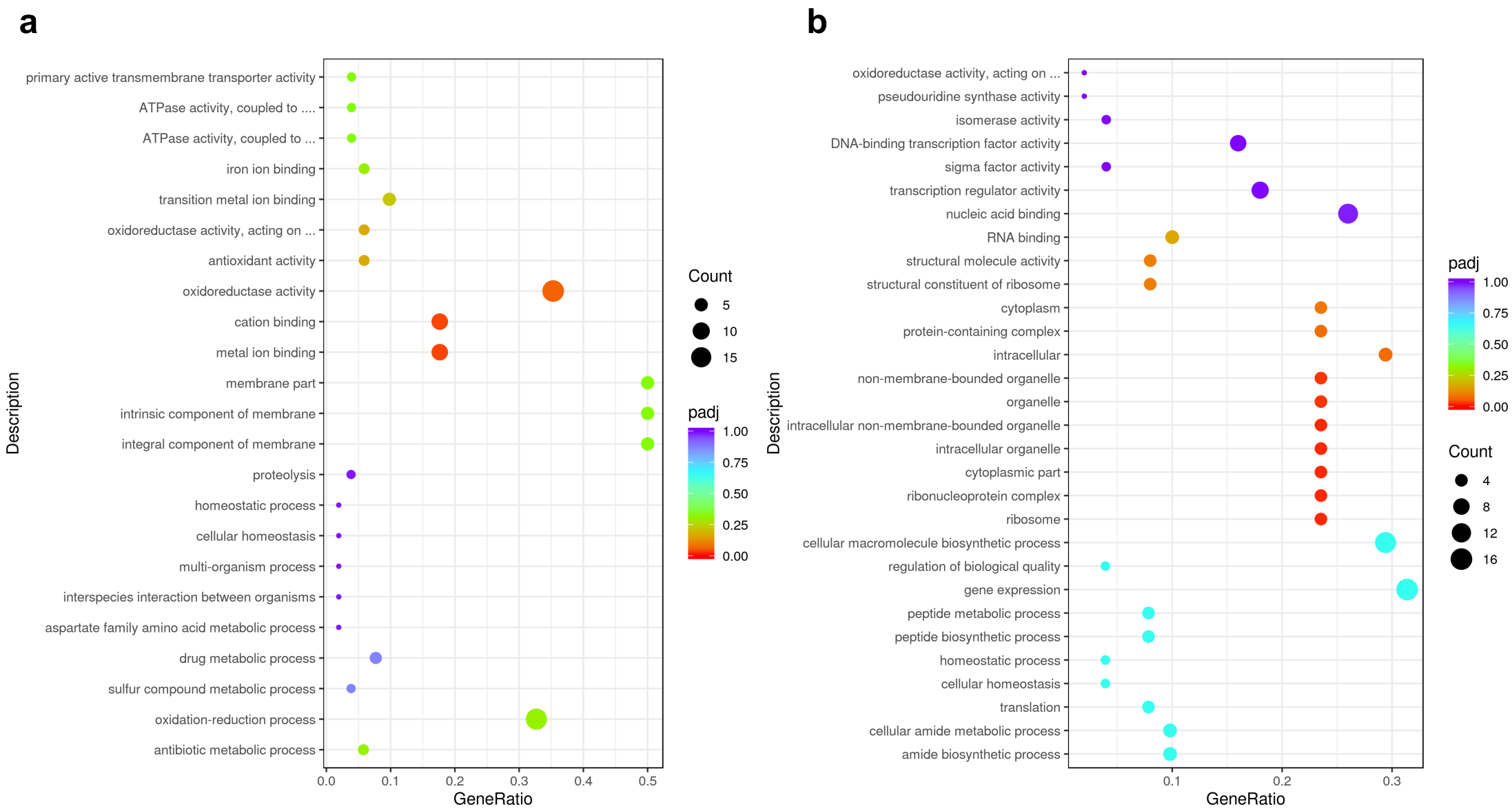

Fig. S5 The mechanism of anti-QS and antibiofilm activity of psoralen. Significantly downregulated (**a**) and upregulated (**b**) GO terms of psoralen-treated PAO1.

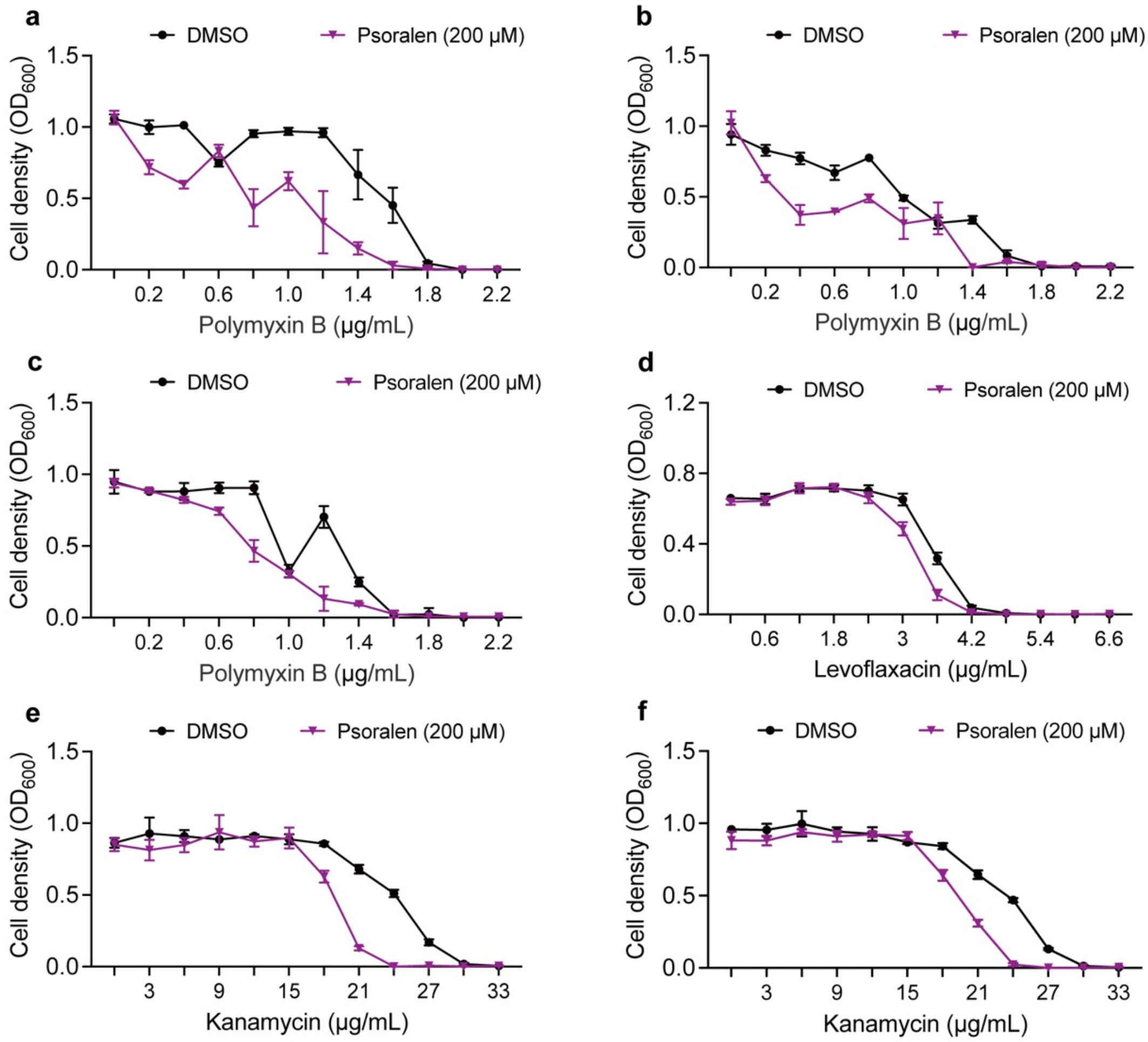

Fig. S6 Effects on growth of clinical isolates combining antibiotics with psoralen. **a** Clinical isolate 3-100-1. **b** Clinical isolate 7-61-28. **c** Clinical isolate 8-61-4B. **d** Clinical isolate 1-65-1. **e** Clinical isolate 3-61-7. **f** Clinical isolate 4-61-8. The results were presented as mean ± SD, and three independent experiments.

Supplementary Tables

Table S1. Docking scores and binding sites of receptors with ligands

Receptors	Ligands	Docking scores (kcal/mol)	H-bonds	Van der waals	π-stacking	Slat bridges
LasR	3-oxo-C12-HSL	-7.80	Trp-60, Thr-75, Thr-115, Ser-129	**Leu-36**, Tyr-47, Ala-50, **Tyr-64**, Ala-70, Val-76, Trp-88	/	/
LasR	Psoralen	-8.02	/	**Leu-36**, Tyr-56, Trp-60, **Tyr-64**, Trp-88, Phe-101, Leu-110	Try-56	Arg-61
RhlR	C4-HSL	-5.46	Asp-81, Ser-135	**Ala-83**, Trp-96, **Phe-101**, Val-133	/	/
RhlR	Psoralen	-6.15	/	Trp-68, Tyr-72, **Ala-83**, **Phe-101**	Tyr-64, Tyr-72, Trp-96	/
PqsR	PQS	-7.62	Ile-236, Ala-237	**Ala-102**, Ala-130, **Ile-149**, **Thr-166**, Ala-168, Leu-197, Leu-208, **Phe-221**, Ile-236, **Pro-238**, Thr-265	/	/
PqsR	Psoralen	-7.09	Ala-168, Thr-265	**Ala-102**, **Ile-149**, **Thr-166**, **Phe-221**, **Pro-238**	/	/

The **bold** indicated the same binding sites.

Table S2. Primers used in this study

Gene	Primer direction	Sequence (5’-3’)	Source
16S RNA	Forward	TCGCATCCTGTTGTCCTCCA	(Yuan et al. 2022)
16S RNA	Reverse	TTAGCCAGGGTCAGCGTCA	
lasR	Forward	CTTCATCGTCGGCAACTAC	(Yuan et al. 2022)
lasR	Reverse	GTCTGGTAGATGGACGGTTC	
lasB	Forward	ATCGGCTACGACATCAAGAAGG	(Yuan et al. 2022)
lasB	Reverse	CCGCTGTTGTAGTTGCTGGTG	
rhlR	Forward	GCTCCTCGGAAATGGTGGT	(Yuan et al. 2022)
rhlR	Reverse	GGAAAGCACGCTGAGCAAAT	
rhlA	Forward	ACTGAACCAGGCGATGCTC	(Yuan et al. 2022)
rhlA	Reverse	GCTCCAGGCAAGCCAAGTA	
pqsR	Forward	CACTGGTTGAAGCGGGAGA	(Yuan et al. 2022)
pqsR	Reverse	TCGTTCTGCGATACGGTGAG	
pqsA	Forward	GCTGAGCGGTCCTTTGGC	(Yuan et al. 2022)
pqsA	Reverse	TGGAACCCGAGGTGTATTGC	
pslA	Forward	TGGGTCTTCAAGTTCCGCTC	This study
pslA	Reverse	ATGCTGGTCTTGCGGATGAA	
pilV	Forward	ACGACGTCAAGGACCAGATG	This study
pilV	Reverse	AGCTGCGGCAGATGTAGTAG	
hcnA	Forward	GCAGACATGACCATCCACCTC	(Yuan et al. 2022)
hcnA	Reverse	CGGTTGCTTTCGGTTTCCA	
phzA1	Forward	GCAACTGGACCACGGAAAG	(Yuan et al. 2022)
phzA1	Reverse	GCACGCAGTTTCTGTATCGG	

Yuan Y, Yang X, Zeng Q, Li H, Fu R, Du L, Liu W, Zhang Y, Zhou X, Chu Y, Zhang X, Zhao K (2022) Repurposing Dimetridazole and Ribavirin to disarm *Pseudomonas aeruginosa* virulence by targeting the quorum sensing system. Front Microbiol 13:1–15. https://doi.org/10.3389/fmicb.2022.978502

Table S3. The MICs of *P. aeruginosa* PAO1 and clinical isolates to commonly used antibiotics (μg/mL).

P. aeruginosa isolates	Meropenem	Aztreonam	Kanamycin	Polymyxin B	Levofloxacin	Piperacillin
PAO1	3	4	16	2	0.4	4
1-65-1	8	16	16	1	5	20
3-61-7	8	16	64	1.5	0.7	96
3-100-1	16	1	16	2.5	2.4	4
4-61-8	8	16	64	1	0.4	96
5-61-8	8	32	64	1.5	0.5	96
5-R5-3A	16	1	32	1	0.5	8
7-61-28	8	8	32	4	0.2	4
8-61-4B	16	16	64	1	0.8	80

Supplementary Dataset

Dataset S1. Significantly differentially expressed genes of psoralen-treated *Pseudomonas aeruginosa* PAO1 at the concentration of 200 μM. $p$adj < 0.05.
